# Allo-Hemodialysis, a Novel Dialytic Treatment Option for Patients with Kidney Failure: Outcomes of Mathematical Modelling, Prototyping, and *Ex Vivo* Testing

**DOI:** 10.3390/toxins16070292

**Published:** 2024-06-26

**Authors:** Vaibhav Maheshwari, Nadja Grobe, Xin Wang, Amrish Patel, Alhaji Cherif, Xia Tao, Joshua Chao, Alexander Heide, Dejan Nikolic, Jiaming Dong, Peter Kotanko

**Affiliations:** 1 Renal Research Institute, 315 East 62nd Street, 3rd Floor, New York, NY 10065, USA; vaibhav.maheshwari@rriny.com (V.M.); nadja.grobe@rriny.com (N.G.); xin.wang@rriny.com (X.W.); amrish.patel29@gmail.com (A.P.); alhaji.cherif@outlook.com (A.C.); tasha.x.tao@gmail.com (X.T.); joshua.e.chao@gmail.com (J.C.); 2 Fresenius Medical Care, 61352 Bad Homburg, Germany; alexander.heide@freseniusmedicalcare.com (A.H.); dejan.nikolic@freseniusmedicalcare.com (D.N.); 3 Fresenius Medical Care, Shanghai 200030, China; aaron.dong@freseniusmedicalcare.com; 4 Icahn School of Medicine at Mount Sinai, New York, NY 10128, USA

**Keywords:** hemodialysis, allo-hemodialysis, alloHD, urea kinetic modeling, acid–base dynamics

## Abstract

It has been estimated that in 2010, over two million patients with end-stage kidney disease may have faced premature death due to a lack of access to affordable renal replacement therapy, mostly dialysis. To address this shortfall in dialytic kidney replacement therapy, we propose a novel, cost-effective, and low-complexity hemodialysis method called allo-hemodialysis (alloHD). With alloHD, instead of conventional hemodialysis, the blood of a patient with kidney failure flows through the dialyzer’s dialysate compartment counter-currently to the blood of a healthy subject (referred to as a “buddy”) flowing through the blood compartment. Along the concentration and hydrostatic pressure gradients, uremic solutes and excess fluid are transferred from the patient to the buddy and subsequently excreted by the healthy kidneys of the buddy. We developed a mathematical model of alloHD to systematically explore dialysis adequacy in terms of weekly standard urea *Kt*/*V*. We showed that in the case of an anuric child (20 kg), four 4 h alloHD sessions are sufficient to attain a weekly standard *Kt*/*V* of >2.0. In the case of an anuric adult patient (70 kg), six 4 h alloHD sessions are necessary. As a next step, we designed and built an alloHD machine prototype that comprises off-the-shelf components. We then used this prototype to perform *ex vivo* experiments to investigate the transport of solutes, including urea, creatinine, and protein-bound uremic retention products, and to quantitate the accuracy and precision of the machine’s ultrafiltration control. These experiments showed that alloHD performed as expected, encouraging future *in vivo* studies in animals with and without kidney failure.

## 1. Introduction

Current hemodialysis (HD) poses significant financial and technical challenges, especially in low- and middle-income countries. A global report has estimated that over two million patients with end-stage kidney disease (ESKD) in 2010 may have experienced premature death due to a lack of access to affordable renal replacement therapy [1]. It is projected that in 2030, 14.5 million people will have ESKD and need treatment, yet only 5.4 million will receive it due to economic, social, and political factors [2]. Conventional HD requires a substantial volume of water (200 to 800 L of tap water per treatment, depending on factors such as dialysate flow rate, treatment time, and rejection rate of the reverse osmosis system), a dialyzer, blood lines, and a sophisticated dialysis machine with pumps, heating, and ultrafiltration control systems to safely deliver the dialysis treatment, mostly in-center. In our research, we systematically examined and challenged long-held HD paradigms, leading to the conceptualization of a novel treatment option that may have the potential to increase access to affordable HD and reduce the loss of human life owing to acute kidney injury (AKI) and ESKD.

When reducing HD to its essentials, we realized that dialysate preparation is a key determinant of dialysis machine complexity, logistics, and environmental impact. This insight was crucial when conceiving allo-hemodialysis (alloHD), a new HD modality. With alloHD, instead of conventional hemodialysate, the blood of a healthy subject (“buddy”) flows through the dialyzer’s blood compartment counter-currently to the patient’s blood flowing through the dialysate compartment. Along the concentration and hydrostatic pressure gradients, uremic solutes and excess fluid are transferred from the patient to the buddy and subsequently excreted by the buddy’s healthy kidneys. Similarly, molecules with a higher concentration on the buddy side (e.g., bicarbonate; water-soluble vitamins) would diffuse into the patient’s blood. In essence, the buddy intermittently “shares” their kidney function with the patient. Because a “dialysate” (i.e., the buddy’s blood) with a physiological chemical composition and temperature is provided by the healthy buddy, the complexity and cost of an alloHD machine are substantially lower compared to a conventional HD machine.

Our research pursued several goals. First, we aimed to explore, through mathematical modeling, the principal ability of alloHD to deliver an adequate dialysis dose; second, we intended to develop and build an alloHD machine prototype; third, we aimed to run *ex vivo* studies to quantify solute kinetics in a bench test setting.

## 2. Results

### 2.1. Mathematical Modeling

**Figure 1:**
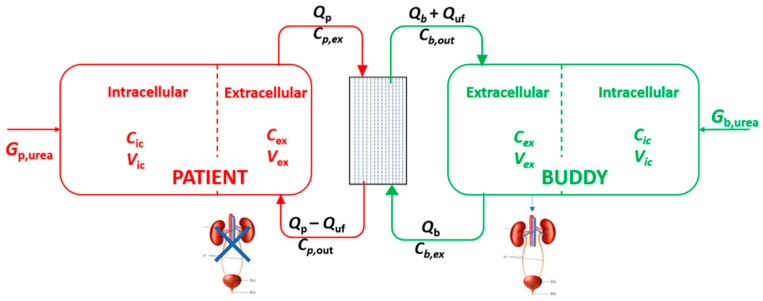
Schematic of the alloHD model where patient and buddy are connected via the dialyzer. The two-compartmental models for both the patient and buddy are shown. The patient is assumed to be anuric, while the buddy’s glomerular filtration rate is set to 120 mL/min. In the schematic, C_ex_ and C_ic_ are the urea concentrations in the extracellular and intracellular fluid volume, respectively; C_p,ex_ and C_b,ex_ are the serum arterial urea concentrations coming out from the patient and buddy, respectively; C_p,out_ and C_b,out_ are the serum urea concentration in the venous (i.e., post-dialyzer) blood stream; V_ex_ and V_ic_ are the extracellular and intracellular fluid volumes, respectively; these are identical to the respective urea distribution volumes; Q_p_ and Q_b_ are the blood flow rates of the patient and the buddy, respectively; Q_uf_ is the ultrafiltration rate; G_p,urea_ and G_b,urea_ are the urea generation rates in the patient and buddy, respectively.

A schematic of the alloHD model is shown in Figure 1. The models of the patient and the buddy comprise extracellular and intracellular compartments. The patient’s and buddy’s extracellular compartments are connected through the dialyzer of the alloHD machine.

While we conducted numerous simulations, we present here only two scenarios: alloHD in an anuric 20 kg child and an anuric 70 kg adult, respectively. Both are allo-hemodialyzed for 4 h against a healthy 70 kg buddy. Urea kinetic modeling (UKM) results are compared to those expected with conventional HD at a dialysate flow rate of 500 mL/min.

#### 2.1.1. Urea Removal and *Kt/V*_urea_

**Figure 2:**
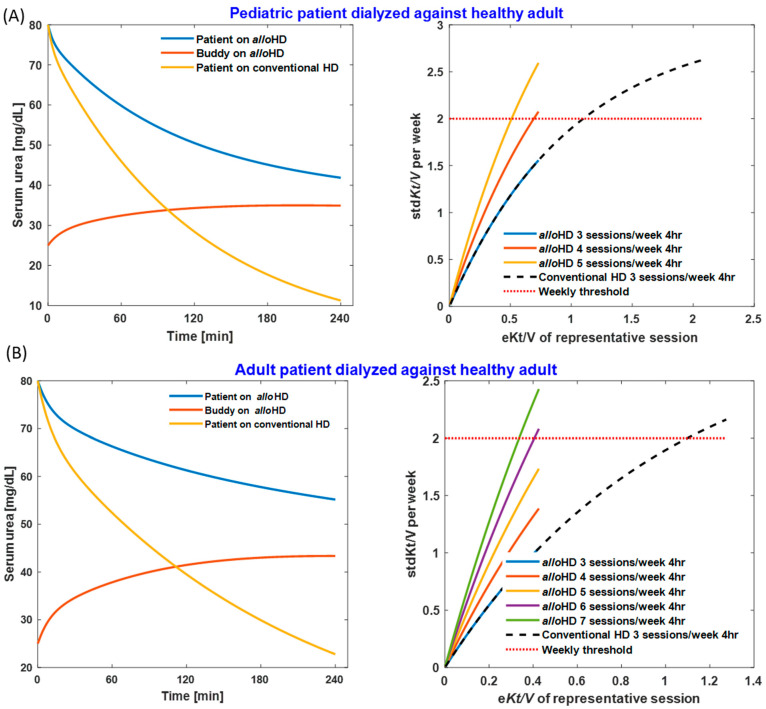
Urea kinetic modeling of alloHD. (**A**) A 20 kg pediatric patient with a Q_b_ of 150 mL/min, an ultrafiltration volume of 1 L, and a dialyzer membrane surface area of 0.9 m^2^. (**B**) A 70 kg adult patient with a Q_b_ of 300 mL/min, an ultrafiltration volume of 2 L, and a dialyzer membrane area of 1.8 m^2^. In both scenarios, a 70 kg healthy adult serves as a buddy with a Q_b_ of 300 mL/min. **Left panel**: The time course of serum urea concentration in alloHD and in conventional HD with a dialysate flow rate of 500 mL/min. **Right panel**: The standard *Kt*/*V* assuming alloHD or conventional HD as a representative session of the week. The pediatric patient’s pre-dialysis weight is 20 kg, with a 14 L urea distribution volume (4.7 L of extracellular fluid and 9.3 L of intracellular fluid). The adult patient’s and buddy’s pre-dialysis weight is 70 kg, with 42 L of urea distribution volume (14 L of extracellular and 28 L of intracellular fluid volume). The dialyzer membrane K_o_A_urea_ is 500 mL/min for the 0.9 m^2^ dialyzer and 1.000 mL/min for the 1.8 m^2^ dialyzer. The intradialytic fluid intake is assumed to be zero for both the patient and buddy.

For the child–adult dyad, the extracorporeal patient blood flow rate is 150 mL/min, and that of the buddy is 300 mL/min; the ultrafiltration volume is 1 L; and the dialyzer surface area is 0.9 m^2^. UKM demonstrates a urea reduction ratio (URR) of 48% using alloHD and 86% using conventional HD, respectively. Four 4 h alloHD sessions are required to attain a weekly standard urea *Kt*/*V* (std*Kt*/*V*) of ≥2.0 (Figure 2A). For the adult–adult dyad, the blood flow rate for both the patient and buddy is 300 mL/min; the ultrafiltration volume is 2 L; and the dialyzer surface area is 1.8 m^2^. AlloHD results in a URR of 31%, compared to 72% with conventional HD. Six 4 h alloHD sessions are necessary to attain a weekly std*Kt*/*V* of ≥2.0 (Figure 2B).

The urea and fluid transferred from the patient to the buddy increase the buddy’s diuresis and, consequently, the flow-dependent renal urea clearance (Supplementary Materials, Figures S1 and S2). Using a model of urine excretion, we estimate that in the child–adult scenario, the buddy excretes 182 mL of additional urine (18% of the 1 L ultrafiltration volume) over 4 h. In the adult–adult scenario, the additional 4 h urine volume amounts to 558 mL (28% of the 2 L ultrafiltration volume).

#### 2.1.2. Correction of Metabolic Acidosis

**Figure 3:**
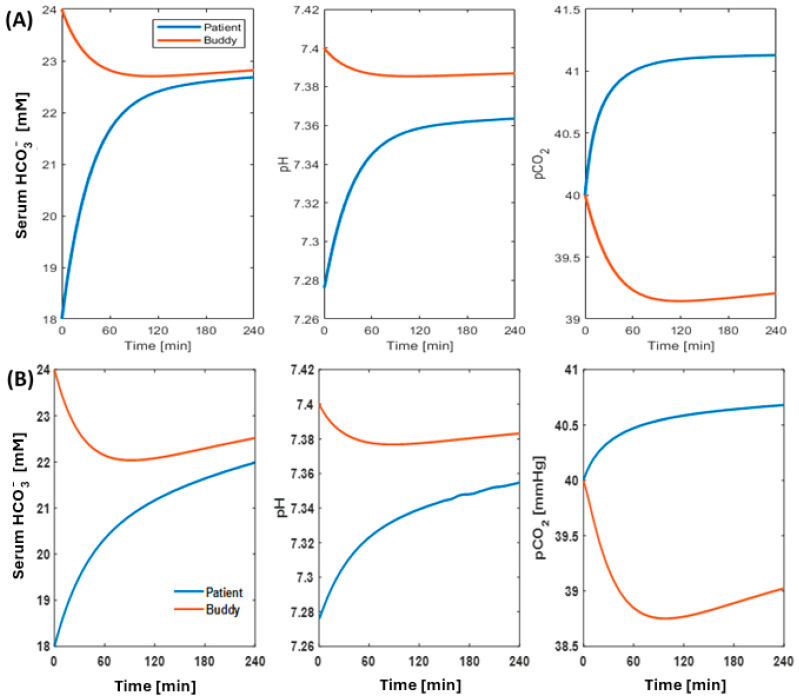
Temporal dynamics of acid–base status in the patient (blue) and the buddy (red) during alloHD: panel (**A**) shows child-patient–adult-buddy dyad and panel (**B**) shows adult–adult dyad. AlloHD setup in individual dyads is same as in Figure 1.

Our simulations indicate that an alloHD session can restore the patient’s acid–base status. The modeling suggests that there is minimal disturbance in the buddy’s acid–base status while providing substantial corrective regulation of the patient’s acid–base homeostasis. Figure 3 shows an example of acid–base dynamics, where alloHD configuration restores the patient’s acid–base status in both the child–adult (see Figure 3A) and adult–adult (see Figure 3B) alloHD scenarios. In the adult–adult pairing, the patient’s HCO_3_^−^ increases from 18 to 22 mM, pH increases to approximately 7.36, and pCO_2_ increases minimally from 40 mmHg to around 40.7 mmHg within the 4 h treatment duration. In the child–adult pairing, similar trends are observed, with the acid–base parameters being slightly above the values seen in the adult–adult pairing. Interestingly, in both scenarios, there are only minimal changes to the buddy’s acid–base status. Since the buddy’s renal function is fully intact, we observe a secondary compensation where HCO_3_^−^, pCO_2_, and pH initially decrease within the first two hours before regulatory compensations attempt to restore the buddy’s acid–base status.

### 2.2. AlloHD Prototyping

#### 2.2.1. AlloHD System Design

**Figure 4:**
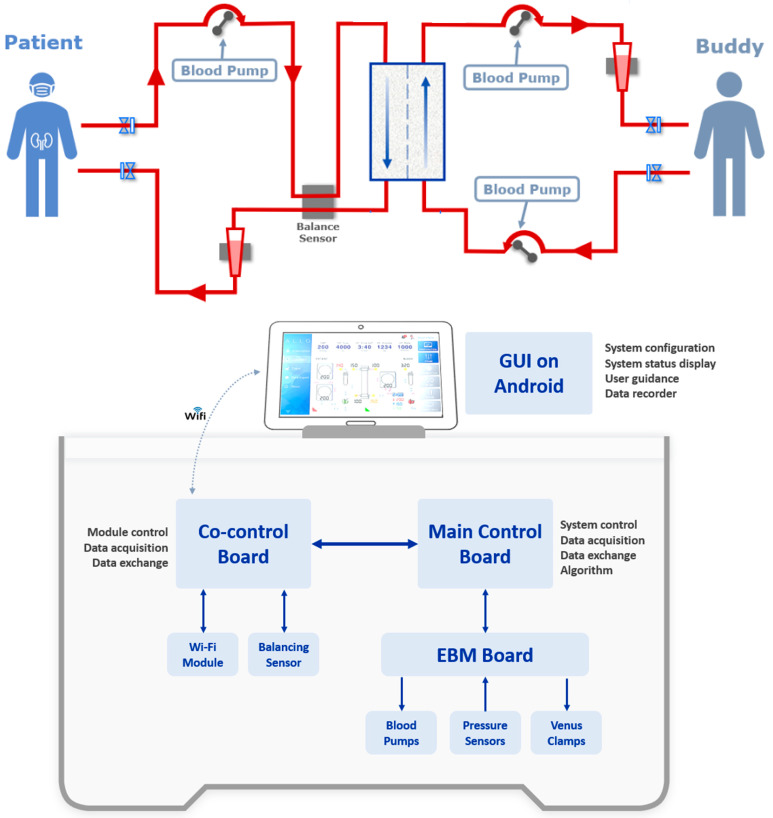
AlloHD system design.

The design of the alloHD machine was driven by the goal of developing a simple, low-complexity, low-cost device that builds on existing off-the-shelf hardware and a graphical user interface (GUI) that can be run on smartphones or tablets. The ultrafiltration control was achieved through an electromagnetic flow (EMF) sensor and three pumps (Figure 4). In the prototype, the alloHD machine was connected via a Wi-Fi signal to a tablet.

The top panel shows the general setup of alloHD, with the patient on the left side and the buddy on the right side. Note that there are three blood pumps in total, one on the patient side and two on the buddy side. The blood flow rate differentials on the buddy-side pumps drive ultrafiltration. 

The bottom panel indicates the software and hardware setup of the alloHD machine (see text for details). The prototype alloHD software has the following modules:

MCU firmware (RTOS based): logic control; blood pump control; ultrafiltration control; read pressure sensor; venous clamp control; data transmission.

CPU software control (Linux based): Wi-Fi communication; balancing sensor control.

GUI software (Android based): system control and setting; tubing connection instruction; system status display; warning message display; treatment data log and trend display; data export.

#### 2.2.2. AlloHD Balancing Technology 

**Figure 5:**
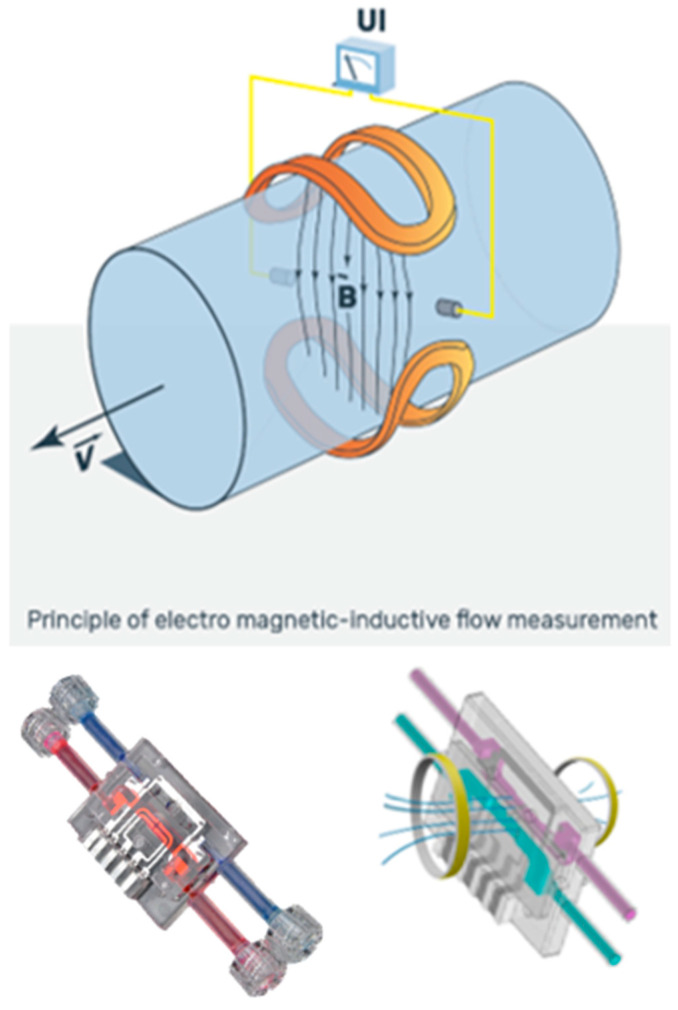
The electromagnetic flow sensor. The top panel shows the principle of electromagnetic-inductive flow measurement. The bottom panel shows the technical realization (see text for details).

Balancing in alloHD was realized with disposable-based technology. The working principle is an EMF sensor, which is well known from existing dialysis machines. However, instead of measuring conventional dialysis fluid, alloHD uses a low-cost single-use sensor on the blood side. Its high level of accuracy is achieved by a double-channel sensor design, where the patient and buddy blood flows enter the same single-use sensor in separate flow channels (Figure 5). A common electromagnetic field penetrates both channels. This design eliminates common mode errors and satisfies the high demands for balancing (i.e., ultrafiltration) accuracy.

#### 2.2.3. AlloHD Machine Prototype 

**Figure 6:**
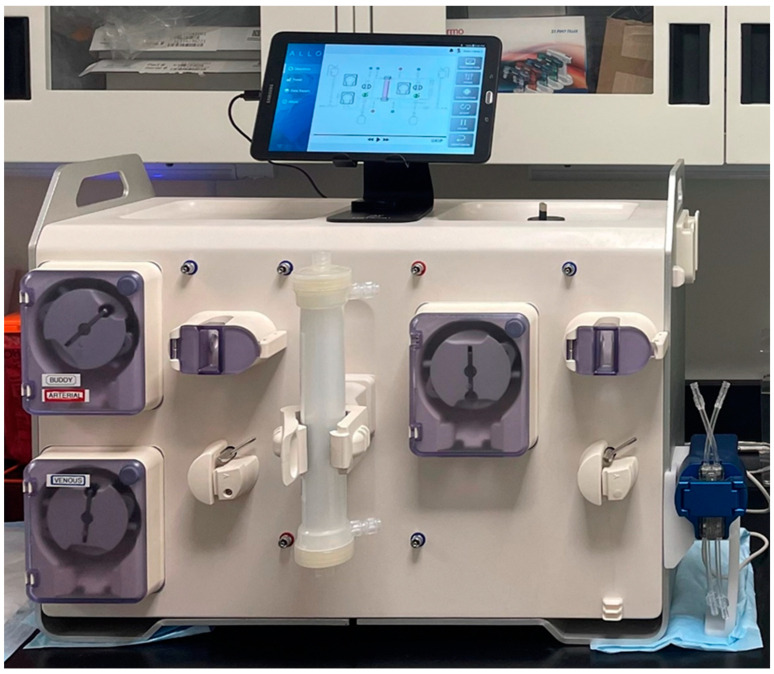
AlloHD prototype. Note the three blood pumps, two on the buddy side (**left**) and one on the patient side (**right**). The alloHD device is connected to a tablet via Wi-Fi. The tablet with a graphical user interface (GUI) is shown on the top.

The prototype of the alloHD machine weighs about 27.6 kg and measures about 64:32:47 cm (length:width:height) (Figure 6).

### 2.3. Bench Tests

**Figure 7:**
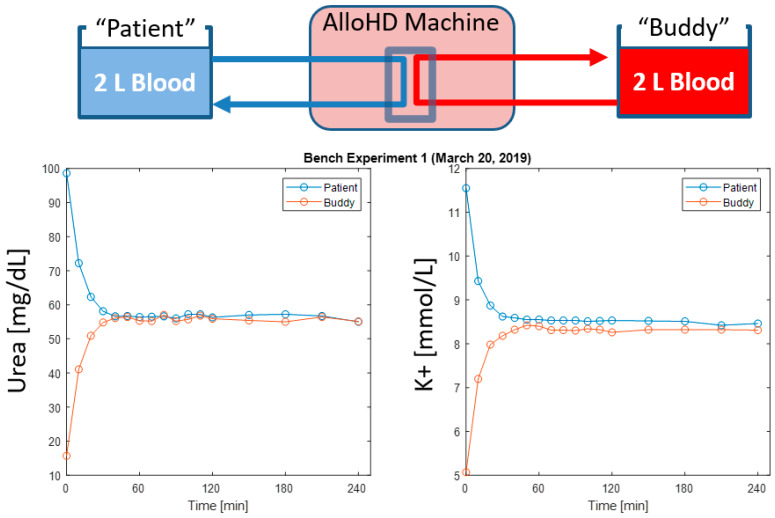
Time course of urea and potassium concentrations in the patient and buddy buckets during alloHD. The patient bucket was spiked with urea and potassium. Note that the equilibration between the patient and buddy buckets is completed after about 30 to 45 min.

To calibrate the urea kinetic model, we conducted *ex vivo* alloHD experiments with human whole blood. The urea concentrations in the patient and buddy buckets equilibrated rapidly (Figure 7). The estimated urea mass transfer coefficient (KoA) was 853 mL/min. Using this estimated KoA, the model-based urea concentrations predicted the observed concentration profiles very well. Also potassium equilibrates rapidly between the two buckets (Figure 7).

**Figure 8:**
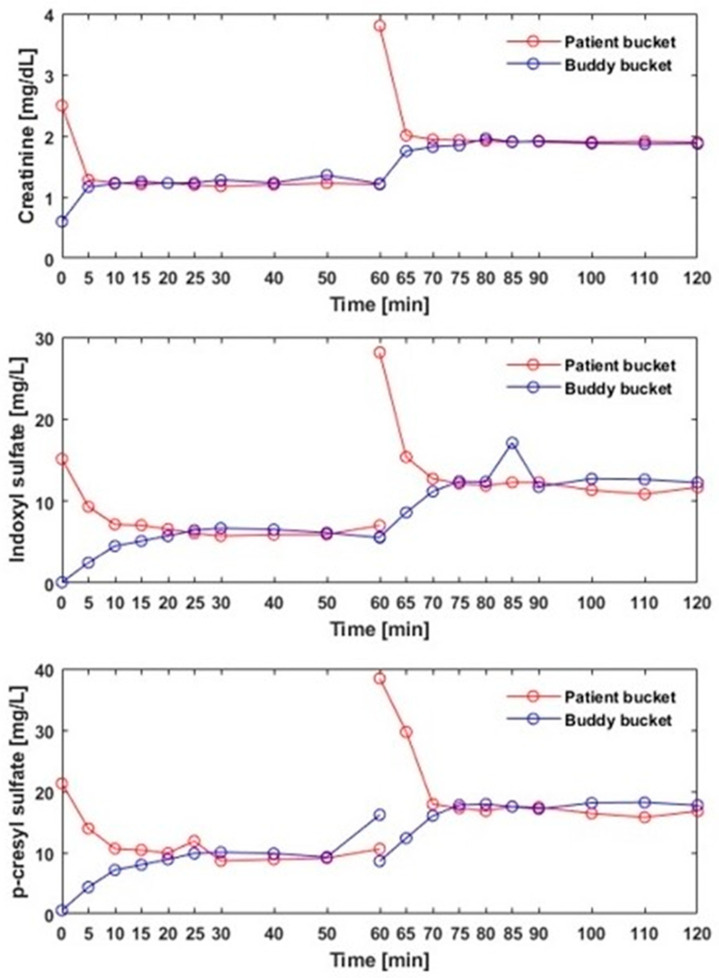
Concentrations of three uremic solutes (creatinine, indoxyl sulfate, and p-cresyl sulfate) over time in the patient and buddy buckets during alloHD. After 60 min, the patient bucket was spiked again with these uremic solutes.

To explore the feasibility of removing creatinine and protein-bound uremic toxins (PBUTs) with alloHD, we conducted an *ex vivo* study with the patient bucket initially spiked with creatinine, indoxyl sulfate (IS), and p-cresyl sulfate (pCS) to establish a diffusion gradient between the patient and buddy. We found that solute concentration differences between the buddy and patient buckets dissipated rapidly (Figure 8). As expected, creatinine concentrations equilibrated faster (within about 5 min), while PBUT concentrations equilibrated more slowly (within 15 to 25 min), presumably due to their protein binding. No blood clots were present even after 2 h of *ex vivo* recirculation.

**Figure 9:**
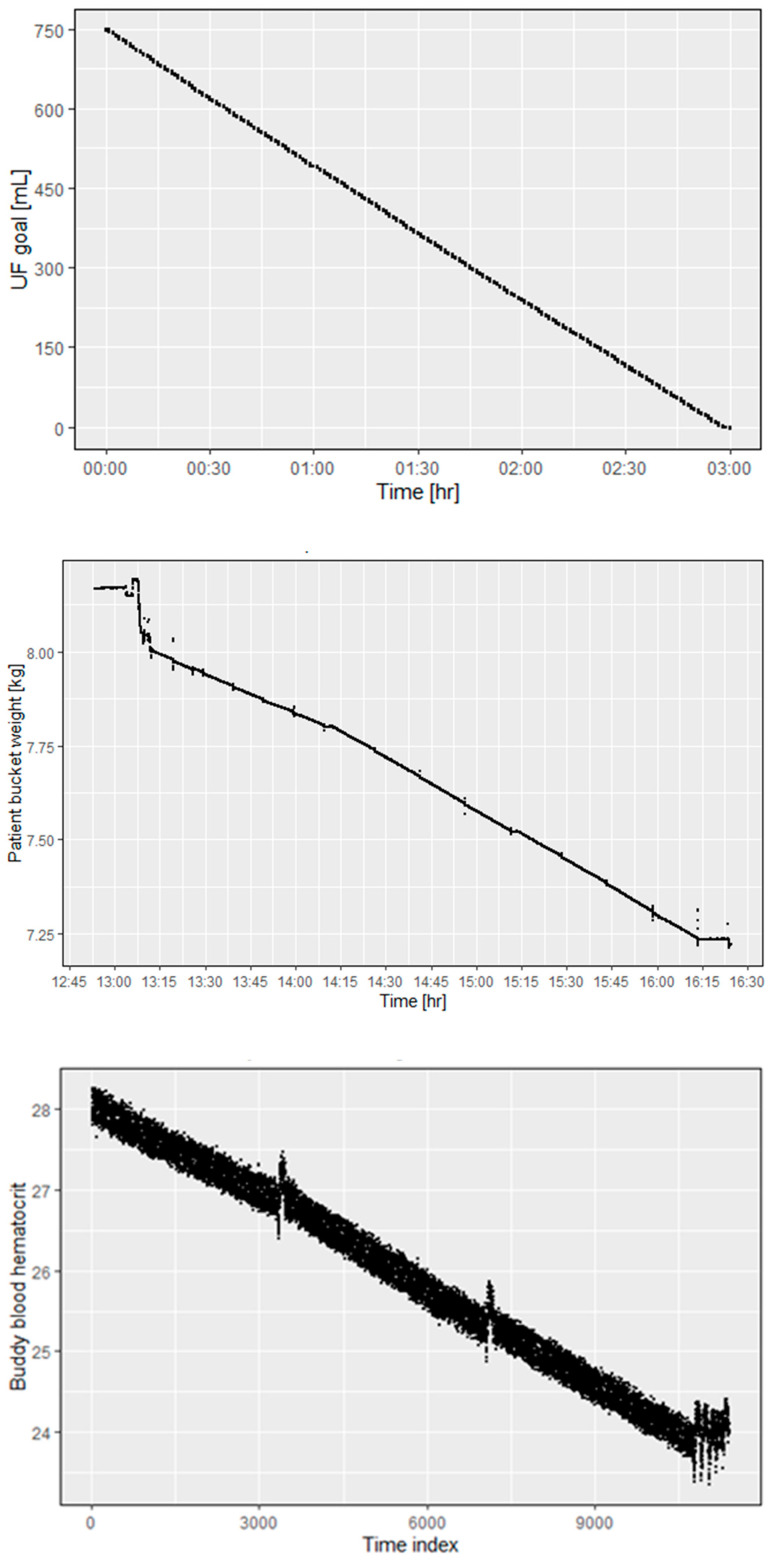
*Ex vivo* ultrafiltration control.

To test the performance of the alloHD prototype’s ultrafiltration control system, we conducted an alloHD experiment using 4 L bovine blood on each side of dialyzer in a 3 h dialysis session. As recorded by the app that controls the ultrafiltration rate, the control system performed as expected. Over the 3 h dialysis period, the set UF goal was achieved in a smooth and linear manner (Figure 9, top panel), which is also indicated by a gradual decrease in the patient bucket weight (Figure 9, middle panel) and declining hematocrit in the buddy blood (Figure 9, bottom panel).

Top panel: The time course of the ultrafiltration volume as per the internal recording. The ultrafiltration goal (750 mL) was reached after 3 h.

Middle panel: The patient bucket weight (~volume) time course during a 3 h alloHD session. The first few minutes of constant volume indicate that the session had not started yet. The initial drop in weight was due to the blood volume replacing the priming fluid volume, which was not recirculated in the bucket. The session started at 13:15 h and ended at 16:15 h. During that 3 h period, the patient bucket lost 0.75 kg.

Bottom panel: Hematocrit measurement using a Crit-Line^®^ (Fresenius Medical Care, Waltham, MA, USA) monitor. Since the buddy blood flows through the lumen, we report the buddy-side hematocrit. The time index is in seconds.

## 3. Discussion

Fundamentally, alloHD resembles the temporary sharing or donation of kidney function between two humans, where a healthy subject (“buddy”) receives fluids and solutes from a patient with kidney failure and subsequently excretes them through their healthy kidneys. As the buddy’s blood functions as the dialysate, the complexity and cost of the envisioned and already prototyped alloHD machine are drastically reduced, making alloHD a viable treatment option for both AKI and ESKD, particularly in resource-limited settings.

Owing to the physiological (i.e., non-zero) levels of urea in the buddy, alloHD provides lower urea clearance than conventional HD. Nevertheless, our urea kinetic modelling show that alloHD provides enough urea clearance to adequately treat an anuric child or adult. Our mathematical model yields essential insights. First, even though urea is continuously transferred, the buddy’s serum urea concentration levels off after an initial increase (Figure 2, left column). While the urea gradient between the patient and buddy decreases, the transferred fluid and urea increase the buddy’s urine flow rate and, hence, the urea clearance. Second, *Q*_b_ is critical for sufficient urea removal, and therefore, chronic alloHD therapy may require central-venous or arterio-venous vascular access, such as an arterio-venous fistula (AVF). Notably, the clinical experience with AVF in patients without kidney failure, such as those with sickle-cell disease [3], hemophilia [4], or home parenteral nutrition [5], is mostly favorable.

Previous dialyzer studies with blood flowing in a dialyzer’s dialysate compartment revealed a reduced coagulation propensity, presumably due to the lower shear stress [6].

The horizontal transfer of viruses between the patient and buddy is a potential risk that needs to be considered. Of note, the pore size of low-flux hollow fiber membranes (1.7–3.5 nm) is well below the size of the smallest viruses known to infect humans (parvovirus, 18–26 nm) and other prevalent viruses (hepatitis B, ~42 nm; hepatitis C, 55–65 nm; human immunodeficiency virus (HIV), 100–160 nm) [7]. Based on the virus geometries relative to the size of the dialyzer membrane pores, viral transfer through an intact membrane is unlikely. However, fiber ruptures may occur. Fortunately, fiber ruptures and blood leaks are rare. Data reported by the U.S. Food and Drug Administration (FDA) between August 2016 and May 2019 in its Manufacturer and User Facility Device Experience (MAUDE) database https://www.accessdata.fda.gov/scripts/cdrh/cfdocs/cfMAUDE/search.CFM (accessed on 30 June 2019) translate into about two to three dialyzer blood leaks per million HD sessions. In alloHD, the detection of blood leaks is a problem. As with any medical intervention, both the patient and buddy need to be made aware of the risks as transparently as possible and tested for prevalent contagious diseases, such as hepatitis and HIV.

AlloHD exposes the buddy to a transient load of uremic solutes. Experience from living kidney donors shows that one healthy kidney can sufficiently clear a person’s solutes. By extension, we posit that the buddy’s two healthy kidneys can excrete intermittent fluid and solute loads. Nevertheless, the buddy’s adaptation to such intermittent fluid and solute loading warrants further clinical studies.

While we focus here on urea kinetics, any solute capable of passing through the dialyzer membrane will diffuse along its concentration gradient between the two counter-current bloodstreams. When the buddy’s bicarbonate level exceeds the patient’s, bicarbonate will diffuse into the patient. It will be important to mathematically model (and eventually study) the transfer of solutes beyond urea and bicarbonate, such as potassium, beta-2 microglobulin, protein-bound uremic toxins, water-soluble vitamins, and drugs. While our urea and acid–base kinetics results indicate that alloHD is a viable dialysis option, studies in large animals (e.g., pigs) are warranted to further explore the biology (e.g., the need for anticoagulation) and technology of alloHD.

An important question is that of the potential acceptance of alloHD by health-care professionals (HCP), patients, and caregivers. Recognizing that alloHD is still hypothetical, Campos et al. surveyed in Morelia, Mexico the acceptance of alloHD in a cross-sectional survey of three groups, caregivers related (rCGs; *N* = 33) and not related (nrCGs; *N* = 42) to ESKD patients, and HCPs (*N* = 50; 39 physicians and 11 non-physicians). The question “Would you donate the function of your kidney intermittently (i.e., serve as an alloHD buddy) to the person you care for?” was answered positively by 76% of rCGs, 67% of nrCGs, and 44% of HCPs. The question “Do you agree with the alloHD procedure?” was answered positively by 88% of rCGs; 91% nrCGs, and 64% HCPs. The question “Would you donate a kidney to the person you care for?” was answered positively by 95% of rCGs, 91% of nrCGs, and 98% of HCPs. This survey indicates that a substantial fraction of caregivers would be willing to serve as an alloHD buddy [8].

In summary, worldwide, every year millions of patients with AKI and ESKD die prematurely due to a lack of access to affordable kidney replacement therapy. We conceptualized, built, and bench-tested alloHD to alleviate the global shortfall in affordable dialysis. Our research indicates that alloHD may indeed work as intended. Studies of allHD in animals with induced AKI are warranted as the next step.

## 4. Materials and Methods

### 4.1. Mathematical Modeling of Solute Transport

#### 4.1.1. Urea Kinetic Modeling (UKM)

To quantify the efficacy of alloHD, we considered two 2-compartment urea models, one each for the patient and the buddy, respectively [9]. Both models were connected with a spatiotemporal dialyzer model (Figure 1) [10]. Following a concentration gradient, urea diffuses across the dialyzer membrane from the patient into the buddy. Our model also accounts for convective urea transfer [11,12].

The buddy received excess fluid and urea from the patient, resulting in increased diuresis. We employed an empirical model to describe the change in urine flow rate as a function of the buddy’s extracellular volume and serum urea concentration (Equation (1)). We segregated the diuretic effects of urea and volume expansion, because, e.g., in a scenario without ultrafiltration, the increase in diuresis depends solely on transferred urea.

(1) $$Urine_{rate}\left(t\right)={Urine}_{baseline}\cdot e^{\beta \left(\frac{{ECV}_{t}-{ECV}_{0}}{{ECV}_{0}}\right)}{\cdot e}^{\gamma \left(\frac{U_{t}-U_{0}}{U_{0}}\right)}$$

${Urine}_{baseline}$ is the buddy’s baseline urine flow rate; ${ECV}_{0}$ and ${ECV}_{t}$ are the extracellular fluid volume at the start of (*t* = 0) and during alloHD, respectively; and $U_{0}$ and $U_{t}$ are the serum urea concentration at the start of (*t* = 0) and during alloHD, respectively.

The diuresis-dependency of urea clearance is given by Equation (2) [13,14].

(2) $${Cl}_{urea}=GFR\cdot e^{-\frac{\alpha }{Urine_{rate}\left(t\right)}}$$

In our simulations we set the buddy’s glomerular filtration rate (GFR) to 120 mL/min.

The empirical models comprised three parameters: a urea clearance saturation parameter (*α*), a factor describing the change in urine flow rate due to fluid gain (*β*), and a factor describing the change in diuresis due to urea gain (*γ*). These parameters were estimated using data from Reid et al., who infused 2 L of Hartmann solution into healthy subjects [15]. The parameters used in our simulations were α = 2.85 mL/min, *β* = 11.02, and *γ* = 0.501, resulting in a baseline urea clearance of 23 mL/min. Using these parameter estimates, we simulated the effect of urea infusion on serum urea concentration and urine volume; the simulation was then compared with data observed in a patient with syndrome of inappropriate ADH secretion who received 240 mL of a 30% urea infusion [16]. For additional details, see Supplementary Materials.

The urea mass transfer rate (KoA) between the intracellular and extracellular compartments was set to 800 mL/min. The initial urea concentrations in the patient and the buddy were set to 80 and 25 mg/dL, respectively. The urea generation rate was set to 10 g/day for the adult patient and the buddy, respectively, and 3.3 g/day in the child. For information regarding the physiological setup of the patient and buddy, see Figure 1 caption. *Q*_b_ was multiplied by 0.894 to obtain the effective urea volumetric flow rate [17]. For the simulated alloHD and conventional HD sessions, we calculated the single-pool and equilibrated *Kt*/*V* [18]. Assuming the simulated session to be representative of the other weekly sessions, we calculated std*Kt*/*V* [19] and aimed for a weekly std*Kt*/*V* of ≥2.0.

#### 4.1.2. Acid–Base Dynamics

To explore the impact of alloHD on both the patient’s and buddy’s acid–base status, we employed a dynamic model of the physiological regulation of the HCO_3_^−^/CO_2_ buffering system developed by Cherif and colleagues [20], configured to describe the coupled transfer between the patient and buddy via alloHD. The model incorporates the production of CO_2_ and H^+^, loss due to non-bicarbonate buffering, and ventilation. In addition, we assumed normal renal function and regulation of HCO_3_^−^/CO_2_ in the buddy, but not in the patient. The patient model was parameterized to yield metabolic acidosis with a pre-dialysis serum bicarbonate of 18 mmol/L. The buddy model was parameterized to physiological acid–base values (see Supplementary Materials for detailed description of the acid–base dynamics).

### 4.2. Prototype Development

#### AlloHD Balancing Technology

Balancing in AlloHD was achieved with a unique disposable-based technology. The working principle is an electromagnetic flow (EMF) sensor, which is well known in existing dialysis machines. But instead of measuring dialysis fluid, alloHD uses a low-cost single-use sensor on the blood side. Its high level of accuracy is achieved by a double channel sensor design where the patient and buddy blood flows enter the same single-use sensor in separate flow channels. A common electromagnetic field penetrates both channels. This design eliminates common mode errors and satisfies the high demand for balancing (ultrafiltration) accuracy (Figure 5). On the buddy side, we used two pumps, and their flow rate differential was used to attain net ultrafiltration.

### 4.3. Ex Vivo Experiments

#### 4.3.1. Urea Kinetics

To calibrate the urea kinetic model, we conducted an *ex vivo* alloHD experiment with human whole blood. We dialyzed a 500 mL patient bucket against a 500 mL buddy bucket for 60 min using a high-flux cellulose triacetate dialyzer (Nipro Cellentia 17H; Nipro Medical Corporation, Bridgewater, NJ, USA) with a surface area of 1.7 m^2^. Heparin (5000 IU/L) was used as anticoagulant. An average blood flow rate of 110 mL/min on both sides was achieved by peristaltic pumps. The patient side was initially spiked with urea to simulate pre-dialysis conditions. Blood samples from both sides were collected at multiple time-points in the experiment. Serum urea was measured using automated spectrophotometry.

#### 4.3.2. Removal of Creatinine and Protein-Bound Uremic Solutes

Two buckets of whole blood (anticoagulated with 5000 IU/L heparin) were designated as patient and buddy and dialyzed against each other for 2 h, with initial flow rates of 110 mL/min for both circuits, using a high-flux cellulose triacetate dialyzer (Nipro Cellentia 17H, surface area 1.7 m^2^) and targeting zero net ultrafiltration. The patient bucket was initially spiked with creatinine, indoxyl sulfate (IS), and p-cresyl sulfate (pCS) to establish a diffusion gradient between the patient and buddy. This was followed by a 2nd spike one hour into the experiment. After each spike, blood samples from both sides were collected every 5 min for 30 min, then every 10 min for the next 30 min. IS and pCS were measured via liquid chromatography–mass spectrometry after liquid–liquid extraction; creatinine was determined via spectrophotometry.

#### 4.3.3. Ultrafiltration Performance

We used 4 L bovine blood on each side of the dialyzer (Nipro Cellentia 17H, surface area 1.7 m^2^). The blood flow rates for the patient and buddy side were set to 150 and 200 mL/min, respectively. The ultrafiltration volume was set to 750 mL over a 3 h dialysis duration.

## Data Availability

Data request should be directed to the corresponding author.

## Supplementary Materials

The following supporting information can be downloaded at: https://www.mdpi.com/article/10.3390/toxins16070292/s1, Figure S1: Effects of 2 L Hartmann solution infusion; Figure S2: Effect of 240 mL of 30% urea infusion in a patient with SIADH; Table S1: Nomenclature table for urea dynamics; Table S2: Additional model variables and parameters for Acid-base dynamics (reference [20,21,22,23,24,25,26,27,28,29,30,31,32,33,34,35,36] are cited in the Supplementary Materials).
