# Supplementary material for: Allo-Hemodialysis, a Novel Dialytic Treatment Option for Patients with Kidney Failure: Outcomes of Mathematical Modelling, Prototyping, and Ex Vivo Testing
Source: Toxins (Basel). 2024 Jun 26;16(7):292. doi: 10.3390/toxins16070292 (PMC11281302; doi:10.3390/toxins16070292)
Supplement: Supplementary file 1 [file toxins-16-00292-s001.zip › toxins-2961265-supplementary.pdf]

SUPPLEMENTARY MATERIAL

Allo-hemodialysis (alloHD) refers to a novel dialysis method where conventional hemodialysis is modified such that the usual dialysate is replaced by circulating blood from a healthy subject ("buddy"). Solutes are transferred from the patient to the buddy by diffusion and convection and excreted by the buddy's functionally healthy kidneys. Diffusion is bidirectional and hence solutes, e.g., bicarbonate, can also move from the buddy to the patient in the presence of a favorable concentration gradient.

To quantitatively examine the alloHD concept, we developed a series of mathematical models of alloHD to explore its effectiveness on patient's and buddy solute and electrolyte kinetics. Here, we focus mainly on urea and acid-base dynamics, in which the healthy buddy temporarily shares their kidney function with an anuric patient.

Mathematical model:

Urea Dynamics:

We consider a two-compartment urea model for both patient and buddy [9]. Unlike the traditional two-compartmental model where dialytic removal is lumped into urea clearance (K_D), we develop a spatiotemporal dialyzer model, where urea diffusion is governed by the membrane mass-transfer coefficient K_oA_{urea}. The model also accounts for convective urea transport in the presence of ultrafiltration [11]. Using this model, we can independently study the effect of blood flow rates on the patient and buddy sides, respectively. Below we present the model equations; the symbols are listed in **Table S1**.

<u>Patient model</u>:

$$\begin{aligned}
\frac{d(U_{p,ex}V_{p,ex})}{dt} &= -Q_{pi}U_{p,ex} + (Q_{pi} - Q_{uf})U_{p,out} + K_{ic}(U_{p,ic} - U_{p,ex}),\\
\frac{d(U_{p,ic}V_{p,ic})}{dt} &= G_{p,urea} - K_{ic}(U_{p,ic} - U_{p,ex}),\\
\frac{dV_{p,ex}}{dt} &= -Q_{uf}\\
\frac{dV_{p,ic}}{dt} &= 0.
\end{aligned} \tag{S1}$$

Dialyzer model:

$$\frac{\partial U_p}{\partial t} = -\frac{1}{N \cdot A}\frac{\partial(Q_p U_p)}{\partial x} - \frac{Pe}{e^{Pe}-1}\frac{1}{N \cdot A \cdot L}\mathrm{K_oA_{urea}}(U_p - U_b) + \frac{1}{N \cdot A}\frac{\partial Q_p}{\partial x}U_p,$$
$$\frac{\partial U_b}{\partial t} = \frac{1}{N \cdot A_b}\frac{\partial(Q_b U_b)}{\partial x} + \frac{Pe}{e^{Pe}-1}\frac{1}{N \cdot A_b \cdot L}\mathrm{K_oA_{urea}}(U_p - U_b) - \frac{1}{N \cdot A_b}\frac{\partial Q_b}{\partial x}U_b,$$
$$Q_p = Q_{pi} - \frac{x}{L}Q_{uf},$$
$$Q_b = Q_{bi} + \frac{L-x}{L}Q_{uf}. \tag{S2}$$

Buddy model:

$$\frac{d(U_{b,ex}V_{b,ex})}{dt} = -Q_{bi}U_{b,ex} + (Q_{bi} + Q_{uf})U_{b,out} + K_{ic}(U_{b,ic} - U_{b,ex}) - Cl_{uera}U_{b,ex},$$
$$\frac{d(U_{b,ic}V_{b,ic})}{dt} = G_{b,urea} - K_{ic}(U_{b,ic} - U_{b,ex}),$$
$$\frac{dV_{b,ex}}{dt} = Q_{uf} - Urine_{rate},$$
$$\frac{dV_{b,ic}}{dt} = 0. \tag{S3}$$

A feature unique to alloHD is the buddy who receives both urea and ultrafiltrate from the patient. Urea acts as an osmotic solute that induces osmotic diuresis and the ultrafiltrate results in volume expansion, so both factors increase the buddy's urine flow rate. We modeled the joint effect of fluid and urea transfer on the buddy's urine flow rate using an empirical relationship that describes the change in urine flow rate from baseline with respect to the change in extracellular volume and serum urea concentration (Equation S4).

$$Urine_{rate}(t) = Urine_{baseline} \cdot e^{\beta\left(\frac{V_{b,ex}-V_{b,ex0}}{V_{b,ex0}}\right)} \cdot e^{\gamma\left(\frac{U_{b,ex}-U_{b,ex0}}{U_{b,ex0}}\right)} \tag{S4}$$

The relationship between urine flow rate and urea clearance has been studied extensively and described by Equation S5 [13]. As can be seen, the urea clearance approaches glomerular filtration rate (GFR) as the urine flow rate increases.

$$Cl_{urea} = GFR \cdot e^{-\frac{\alpha}{Urine_{rate}(t)}} \tag{S5}$$

The urine flow model comprises two parameters (β, γ) and the urea clearance model one (α). To estimate these parameters, we used data from Reid *et al.*, who infused 2 L of Hartmann Ringer solution into 9 healthy subjects over 1 hour; thereafter the subjects were followed for 5 hours. Subjects refrained from drinking and eating during the study

[15]. We extracted the mean weight loss (due to urine excretion) and urea concentration data to calibrate the buddy model. We assumed a GFR of 120 mL/min in these healthy subjects and that the Hartmann solution distributes into the extracellular compartment only. The estimated parameters (α = 2.85 mL/min, β = 11.02, γ = 0.501) resulted in a baseline urine flow rate of 1.7 mL/min. The model fits are presented in **Figure S1**.

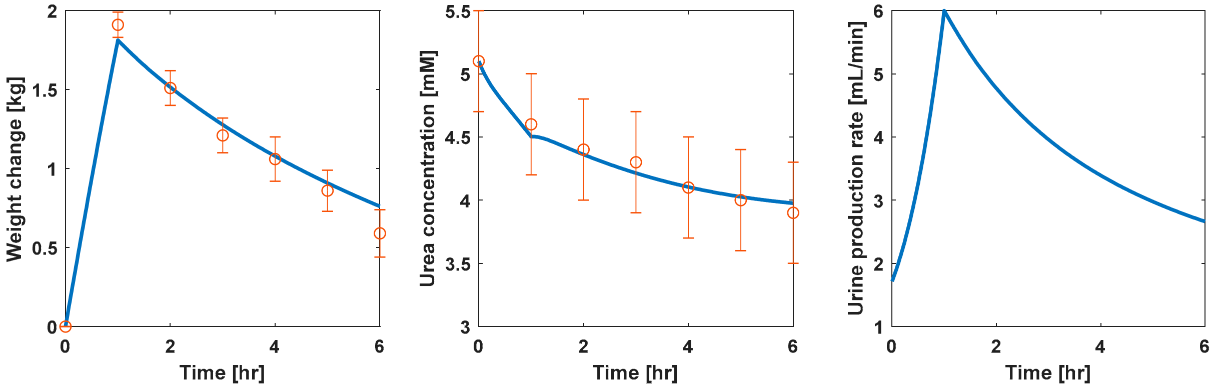

Figure S1: Effects of 2 L Hartmann solution infusion between time 0 and 1 hour on weight (left panel) and serum urea concentration (middle panel) in nine healthy subjects; the observed data are shown in red, the circles represent the means and the whiskers standard deviations. The model fits are shown in blue lines. The predicted effect of Hartmann solution infusion on urine production rate is presented on the right panel. The observed data are extracted from Reid *et al*. [15].

Note that the Hartmann solution closely resembles serum water and can therefore be used to approximate the patient's ultrafiltrate. However, the infusion of Hartmann solution does not capture the effect of urea infusion on urine flow. To model the effect of urea infusion on urine flow rate, we resorted to observations made in subjects who received urea infusions. We could not locate reports quantifying in healthy subjects the effects of urea infusion on urea levels and urine flow rates. Absent such data, we utilized data from a hyponatremic patient with syndrome of inappropriate ADH secretion (SIADH) reported by Decaux *et al*. The authors infused 240 mL of 30% urea in saline. The infusion was given over a period of 6 hours, the patient was observed for 32 hours [16]. Urine volumes were collected during 8-hour periods and the serum urea concentrations were measured every 8 hours. To reflect in our model simulations the low pre-infusion urinary flow rate seen in this SIADH patient, we set the parameter α to 1.5 mL/min; GFR, β, and γ

remained the same. As can be seen, the model captures urea levels and urine volumes in a qualitatively satisfying way (**Figure S2**). Of note, the observed urea levels and urine flow rates are higher in the patients compared to the model predictions. We consider this not surprising because it is safe to assume that the patients had some food and fluid intake during the 32-hour observation period.

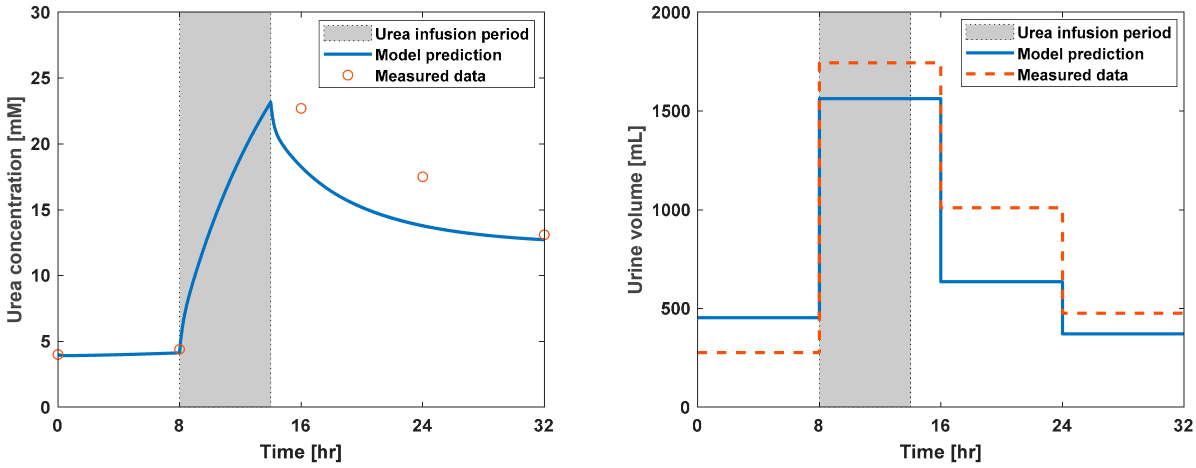

Figure S2: Effect of 240 mL of 30% urea infusion in a patient with SIADH. The infusion took place between hours 8 and 14 (shaded gray). The observed (red symbols) and predicted (blue symbols) urea levels (left panel) and urine volumes (right panel) are shown. In the simulations the GFR was set to 120 mL/min. While not reported by *Decaux et al.*[16], we consider it very likely that the patient ate and drank during the 32-hour observation period, resulting in higher than predicted urine volumes and urea levels.

Acid-Base Dynamics:

We modified the acid-base homeostasis model developed by Cherif and colleagues, which describes the extracellular concentration of hydrogen ion, bicarbonate, and carbon dioxide in the patient [21–24]. The dialyzer model of Maheshwari and colleagues is adapted to reflect our specific application [25]. The model schematics is shown in the main text (see Figure 1).

<u>Patient models</u>

A dynamic model of physiological regulation of HCO_3^-/CO_2 buffering system with Henderson-Hasselbalch mass-action kinetics is used to describe a coupled transfer between patient and buddy via alloHD. The model incorporates

endogenous production of CO_2 and H^+ due to cellular metabolic activities (captured by P_{CO_2} and P_{H^+} for CO_2 and H^+, respectively), loss due to non-bicarbonate buffering such as phosphate (presented by $\gamma_{H^+}C_{H^+}$, where γ_{H^+} is the mobilization or removal rate), physiologic regular of HCO_3^-/CO_2 through ventilation and/or renal excretion (for healthy buddy only). That is, under physiologic condition, pH is regulated mainly by 3 mechanisms: (1) chemical acid-base buffering modeled by Henderson-Hasselbalch mass-action kinetics with K_{H^+,HCO_3^-} and K_{CO_2} representing the association and disassociate rates, respectively; (2) renal filtration and (3) respiratory control by lung. In normal physiologic condition, kidney regulates HCO_3^- level by either secreting excess H^+ into tubular lumen during metabolic acidosis, or excretes or reabsorbs HCO_3^- in the proximal and distal segments of nephrons [26–29,36,37]. For buddy, this is described by the renal filtration term $\phi_{CO_2}\,C_{CO_2} - D_{HCO_3^-}C_{HCO_3^-}$. For the patient, the renal function is impaired and is replaced using dialyzer, in particular by dialyzer fluxes. The expression, $-Q_pC_{HCO_3^-}$ and $\left(Q_p - Q_{uf}\right)C_{HCO_3^-,out}$ account for bicarbonate flux from the patient and post-dialyzer flux to the patient, respectively, where Q_p, is the blood flow rate, and Q_{uf} is the ultrafiltrate rate. The lung regulates CO_2 removal by increasing or decreasing ventilation, which is triggered by the response of central and peripheral chemoreceptors to changes in pCO_2. Here, $D_{CO_2}\mathrm{V}_0Y_{CO_2}$ describes removal of CO_2 through ventilation by lung, which is usually characterized by blood volume, cardiac output, arteriovenous difference of CO_2, where $D_{CO_2}$and V_0 ventilation rate and minute ventilation, respectively. Under the above assumptions and regulatory mechanisms [26,27,29–35], the dynamic model equations for the acid-base system is as follows (see Table S2):

$$\frac{d\left(C_{p,H^+}V_{p,ex}\right)}{dt} = \underbrace{P_{p,H^+}}_{Endogenous\ H^+\ production} - \underbrace{\gamma_{p,H^+}C_{p,H^+}}_{Lumped\ non-bicarbonate} - \underbrace{\left(K_{H^+,HCO_3^-}C_{p,H^+}C_{p,HCO_3^-} + K_{CO_2}C_{p,CO_2}\right)V_{p,ex}}_{Henderson-Hasselbach\ kinetics},$$

$$\frac{d\left(C_{p,HCO_3^-}V_{p,ex}\right)}{dt} = \underbrace{-Q_pC_{p,HCO_3^-}}_{Flux\ from\ patient} + \underbrace{\left(Q_p - Q_{uf}\right)C_{p,HCO_3^-,out}}_{Post-dialyzer\ flux\ to\ patient} - \underbrace{\left(K_{H^+,HCO_3^-}C_{p,H^+}C_{p,HCO_3^-} + K_{CO_2}C_{p,CO_2}\right)V_{p,ex}}_{Henderson-Hasselbach\ kinetics},$$

$$\frac{d\left(C_{p,CO_2}V_{p,ex}\right)}{dt} = \underbrace{P_{p,CO_2}}_{Endogenous\ CO_2\ production} - \underbrace{D_{p,CO_2}\mathrm{V}_{p,0}C_{p,CO_2}}_{Respiratory\ ventilation} + \underbrace{\left(K_{H^+,HCO_3^-}C_{H^+}C_{HCO_3^-} + K_{CO_2}C_{CO_2}\right)V_{p,ex}}_{Henderson-Hasselbach\ kinetics}.$$

During dialysis, a patient loses a significant amount of fluid, which is assumed to be done at a constant ultrafiltration rate, Q_{uf}, and fluid removal by ultrafiltration occurs in proportion to the compartmental distribution volume, where the extracellular fluid volume is given as follows:

$$\frac{dV_{ex}}{dt} = \underbrace{-Q_{uf}}_{Ultrafiltration\ rate}$$

Dialyzer models:

The dialyzer model consists of two spatial temporal models describing both patient and buddy sides using hyperbolic partial differential equations. The equation depicting the concentration of HCO_3^- in the patient side is as follows:

$$\frac{\partial c_{p,HCO_3^-}}{\partial t} = \underbrace{-\frac{1}{N \cdot A}\frac{\partial\left(Q_p c_{p,HCO_3^-}\right)}{\partial x}}_{Axial\ convection} + \underbrace{\frac{1}{N \cdot A}\frac{\partial Q_p}{\partial x} c_{p,HCO_3^-}\left(1 - \sigma_{c_{HCO_3^-}}\right)}_{Radial\ convection} - \underbrace{\frac{Pe}{e^{Pe} - 1}\frac{1}{N \cdot A \cdot L} K_o A\left(c_{p,HCO_3^-} - \frac{c_{b,HCO_3^-}}{\beta}\right)}_{Gibbs-Donnan\ corrected\ effective\ diffusion},$$

where N is the number of fibers, A denotes fiber cross-sectional area, $\sigma_{c_{HCO_3^-}}$ represents the HCO_3^- reflection coefficient, $Pe = \frac{\left(1-\sigma_{c_{HCO_3^-}}\right)Q_{uf}}{K_o A}$ defines the Peclet, L is the fiber length, $K_o A$ is the effective membrane mass-transfer coefficient for HCO_3^-, β is Gibbs-Donnan correction factor and is set to a constant value of 1.05 which corresponds to 5% of HCO_3^-. For the buddy side, we assume that the buddy blood flow is uniform and equally shared by the N fibers present in the dialyzer housing. The hyperbolic partial differential equation is as follows:

$$\frac{\partial c_{b,HCO_3^-}}{\partial t} = \underbrace{\frac{1}{N \cdot A_b}\frac{\partial\left(Q_b, c_{b,HCO_3^-}\right)}{\partial x}}_{Axial\ convection} - \underbrace{\frac{1}{N \cdot A_b}\frac{\partial Q_b}{\partial x} c_{HCO_3^-}\left(1 - \sigma_{c_{HCO_3^-}}\right)}_{Radial\ convection} + \underbrace{\frac{Pe}{e^{Pe} - 1}\frac{1}{N \cdot A_b \cdot L} K_o A\left(c_{p,HCO_3^-} - \frac{c_{b,HCO_3^-}}{\beta}\right)}_{Gibbs-Donnan\ corrected\ effective\ diffusion}$$

where A_d circular cross-sectional area of annulus space for buddy blood flow around a fiber. In addition, blood flow rate (Q_p) decreases along the fiber length in the dialyzer due to ultrafiltration, and it is assumed to decrease linearly along the fiber length. Similarly, the buddy's blood flow rate increases by the amount of fluid removed by ultrafiltration from the blood side to the dialysate side, resulting in counter-current kinetics. The spatial aspects of blood and dialysate flow rates are shown as follows:

$$Q_p = Q_{pi} - \frac{x}{L} Q_{uf},$$

$$Q_b = Q_{bi} + \frac{(L - x)}{L} Q_{uf}$$

where Q_{pi} and Q_{di} are initial blood flow rates for both patient and buddy. The above model is also used for urea alloHD, where subscripts for HCO_3^- is replaced with "u" for urea.

Buddy models

Acid-Base Dynamics:

In the model for buddy is the same as the patient model with the exception of the inclusion of renal physiologic regulation, which was excluded in the patient model. We assume a normal renal function and its regulation of HCO_3^- /CO_2 in the buddy, but not in the patient. The dynamic model is as follows:

$$\frac{d\left(C_{b,H^+}V_{b,ex}\right)}{dt} = \underbrace{P_{b,H^+}}_{Endogenous\ H^+\ production} - \underbrace{\gamma_{b,H^+}C_{b,H^+}}_{Lumped\ non-bicarbonate} - \underbrace{\left(K_{H^+,HCO_3^-}C_{b,H^+}C_{b,HCO_3^-} + K_{CO_2}C_{b,CO_2}\right)V_{b,ex}}_{Henderson-Hasselbach\ kinetics},$$

$$\frac{d\left(C_{b,HCO_3^-}V_{b,ex}\right)}{dt} = \underbrace{\phi_{b,CO_2}C_{b,CO_2} - D_{b,HCO_3^-}C_{b,HCO_3^-}}_{Renal\ filtration} + \underbrace{-Q_pC_{b,HCO_3^-}}_{Flux\ from\ patient}$$

$$+ \underbrace{\left(Q_p - Q_{uf}\right)C_{b,HCO_3^-,out}}_{Post-dialyzer\ flux\ to\ patient} - \underbrace{\left(K_{H^+,HCO_3^-}C_{b,H^+}C_{b,HCO_3^-} + K_{CO_2}C_{b,CO_2}\right)V_{b,ex}}_{Henderson-Hasselbach\ kinetics},$$

$$\frac{d\left(C_{b,CO_2}V_{b,ex}\right)}{dt} = \underbrace{P_{b,CO_2}}_{Endogenous\ CO_2\ production} - \underbrace{D_{b,CO_2}\mathrm{V}_{\mathrm{b},0}C_{b,CO_2}}_{Respiratory\ ventilation} + \underbrace{\left(K_{H^+,HCO_3^-}C_{b,H^+}C_{b,HCO_3^-} + K_{CO_2}C_{b,CO_2}\right)V_{b,ex}}_{Henderson-Hasselbach\ kinetics}.$$

$$\frac{dV_{b,ex}}{dt} = \underbrace{Q_{uf}}_{Ultrafiltration\ rate}$$

The patient model is parameterized to yield various degrees of metabolic acidosis, while the buddy model is parameterized to physiological values.

Table S1: Nomenclature table for urea dynamics

Symbol	Description	Unit
A	Fiber cross-sectional area through which patient blood flows	m^2
A_b	Interstitial space cross-sectional area around a fiber through which buddy blood flows	m^2
Cl_{uera}	Native urea clearance in buddy	mL/min
GFR	Glomerular filtration rate in buddy	mL/min
$G_{p/b,urea}$	Urea generation rate in patient/buddy	gram/day
K_{ic}	Urea mass-transfer coefficient between intracellular and extracellular compartment	mL/min
$\mathrm{K_oA_{urea}}$	Urea mass transfer coefficient of hollow fiber membrane	mL/min
L	Length of fiber/dialyzer	m

N	Number of hollow fibers	–
Pe	Peclet number	–
Q_{bi}	Blood flow rate from buddy to dialyzer (counter-current to patient blood flow)	mL/min
Q_b	Buddy blood flow rate around the hollow fiber in the dialyzer (increasing in counter-current to patient blood flow)	mL/min
Q_p	Patient blood flow rate inside the hollow fiber in the dialyzer (decreasing due to ultrafiltration)	mL/min
Q_{bi}	Blood flow rate from buddy to dialyzer	mL/min
Q_{pi}	Blood flow rate from patient to dialyzer	mL/min
$U_{b,ex0}$	Urea concentration in buddy at the start of alloHD	M
$U_{p/b,ex}$	Urea concentration in patient/buddy extracellular compartment	M
$U_{p/b,ic}$	Urea concentration in patient/buddy intracellular compartment	M
$U_{p/b,out}$	Urea concentration in patient/buddy blood stream leaving the dialyzer	M
$Urine_{baseline}$	Baseline urine production rate in buddy	mL/min
$Urine_{rate}$	Urine production rate in buddy	mL/min
Q_{uf}	Ultrafiltration rate	mL/min
$V_{p/b,ex}$	Extracellular fluid volume in patient/buddy	L
$V_{b,ex0}$	Extracellular fluid volume in buddy at the start of alloHD	L
$V_{p/b,ic}$	Intracellular fluid volume in patient/buddy	L
	Urea clearance saturation parameter	mL/min
β	Factor describing the change in urine flow rate due to fluid gain	–
γ	Factor describing the change in diuresis due to urea gain	–

Table S2: Additional model variables and parameters for Acid-Base dynamics

Symbol	Description	Units
C_{x,H^+}	Concentration of H^+ in the patient (x=p) or buddy (x=b)	M
C_{x,HCO_3^-}	Concentration of HCO_3^- in the patient or buddy	M
C_{x,CO_2}	Concentration of CO_2 in the patient or buddy	M
$V_{x,ex}$	Extracellular volume	L
c_{x,HCO_3^-}	Concentration of dialyzer HCO_3^- in the blood side	M
c_{b,HCO_3^-}	Concentration of dialyzer HCO_3^- in the buddy side	M
$c_{x,HCO_3^-,out}$	Concentration of HCO_3^- at dialyzer exit	M
P_{x,H^+}	Endogenous production of H^+	$mol \cdot min^{-1}$
P_{x,CO_2}	Endogenous production of CO_2	$mol \cdot min^{-1}$
γ_{x,H^+}	Non-bicarbonate removal or mobilization of H^+	$L \cdot min^{-1}$
$D_{x,CO_2}V_{x,0}$	Respiratory rate	$L \cdot min^{-1}$
K_{H^+,HCO_3^-}	Hydration reaction/association rate	$M^{-1}\ min^{-1}$
K_{CO_2}	Dehydration reaction/dissociation rate	min^{-1}
β	Gibbs-Donnan correction factor	–
$\sigma_{c_{HCO_3^-}}$	Reflection coefficient of concentration species in the serum	–

*Nomenclature with description of symbols, corresponding units and values.
#Calculated for each patient
